# Resolving breast cancer heterogeneity by searching reliable protein cancer biomarkers in the breast fluid secretome

**DOI:** 10.1186/1471-2407-13-344

**Published:** 2013-07-12

**Authors:** Ferdinando Mannello, Daniela Ligi

**Affiliations:** 1 Department of Biomolecular Sciences, Section of Clinical Biochemistry and Cell Biology, University “Carlo Bo”, Urbino, Italy

## Abstract

**Background:**

One of the major goals in cancer research is to find and evaluate the early presence of biomarkers in human fluids and tissues. To resolve the complex cell heterogeneity of a tumor mass, it will be useful to characterize the intricate biomolecular composition of tumor microenvironment (the so called cancer secretome), validating secreted proteins as early biomarkers of cancer initiation and progression. This approach is not broadly applicable because of the paucity of well validated and FDA-approved biomarkers and because most of the candidate biomarkers are mainly organ-specific rather than tumor-specific. For these reasons, there is an urgent need to identify and validate a panel of biomarker combinations for early detection of human tumors. This is especially important for breast cancer, the cancer spread most worldwide among women. It is well known that patients with early diagnosed breast cancer live longer, require less extensive treatment and fare better than patients with more aggressive and/or advanced disease.

**Results:**

In the frame of searching breast cancer biomarkers (especially using nipple aspirate fluid mirroring breast microenvironment), studies have highlighted an optimal combination of well-known biomarkers: uPA + PAI-1 + TF. When individually investigated they did not show perfect accuracy in predicting the presence of breast cancer, whereas the triple combination has been demonstrated to be highly predictive of pre-cancer and/or cancerous conditions, approaching 97-100% accuracy.

**Conclusion:**

Despite the heterogeneous composition of breast cancer and the difficulties to find specific breast cancer biomolecules, the noninvasive analysis of the nipple aspirate fluid secretome may significantly improve the discovery of promising biomarkers, helping also the differentiation among benign and invasive breast diseases, opening new frontiers in early oncoproteomics.

## Review

### Breast tumour heterogeneity, cancer origin and secretome biomarkers

Growing evidence suggests that human cancers develop via a non-linear multi-step process of cellular diversification and evolution. In particular, breast cancer initiation/progression from ductal/lobular system are dynamic processes of cell clonal adaptation to a fluctuating tumour microenvironment [1]. During tumour expansion there is a constant acquisition of genetic and epigenetic alterations, increasing the intra-tumor heterogeneity, and making difficult the development of effective therapies [2].

Recently, the classical hypothesis on the origin of human cancer known as clonal evolution (i.e., “*reiterative cycles of clonal expansion, genetic diversification and clonal selection within the adaptive landscapes of tissue ecosystems*” [3]) has been revisited by the novel stemming tumor evolution model, in which the continuous clonal expansion of tumor cells is both triggered and promoted by additional mutations and guided by Cancer Stem Cells (CSC) (i.e., “*able to evolve as a cancer grows and repopulate the cancer when the bulk of the tumor is wiped out by anticancer drugs*” [4]).

About 150 years after Virchow’s original theory of cancer cell biology (“*tumours as originating from immature cells*” [5]), and half a century after the introduction of the term CSCs (“*a rare subpopulation of multipotent progenitor cells with self-renewal ability different from the bulk cells*” [6]), the effective existence of CSC has been finally demonstrated for the first time in different cancer models (e.g., intestinal [7], brain [8], and skin [9] mouse tumors).

Through different technologically innovative bio-molecular approaches, studies unequivocally demonstrated the cellular heterogeneity of tumors, composed of different set of cells (e.g., differentiated cancer cells, cancer stem cells, non-cancer stem cells and non cancer cells), hierarchically organized and characterized by specific biomolecular and morphological profiles.

It has been clearly demonstrated that cellular heterogeneity is closely related to stochastic transcriptional events, leading to variations in patterns of expression among genetically identical single cells [10]. This cell heterogeneity provides a means for responding to the continuing changes in the microenvironment. So, single cells can easily take advantage of the inherent stochastic variability in gene expression to increase their survival at the expense of the rest of the clonal cell population [11].

Conventional cancer diagnostic tools (such as imaging techniques, biopsies, etc.) are limited by the impossibility to discern the intra-tumour cancer cells heterogeneity. The low sensitivity and specificity of standard methods to detect cancer cells or their specific secreted biomolecules represent one of the major obstacle for cancer diagnosis [10]. In fact, cancer cell heterogeneity may limit (or at least mask) the detection of biomolecules identified only from the averages of a large population of cells, missing (or at least neglecting) molecules produced only from rare cells (such as invasive/metastatizing cancer and/or cancer stem cells) [12].

Through the analytical technique of *single-cell analysis* it is now possible to identify, quantify, isolate, and characterize the heterogenous composition of a tumour mass with single-cell resolution, with high efficiency of cell viability and integrity for genomic, transcriptomic and metabolomic analyses downstream [10]. This system offers several advantages linked to the deeper comprehension of cellular and molecular composition of the cancer mass, because of the possibility to highlight the peculiarity of cellular morphology, whole-genome and whole-gene expression profiles, etc. [13].

Besides the numerous differences detected among cancer cells within a tumour, cancer cell heterogeneity is also actively guided by the surrounding stroma and the components of the cancer microenvironment (e.g., constitutive and criptic biomolecules). In this respect, both cellular and non-cellular components (e.g., fibroblasts, immunocytes as well as structural proteins and extracellular compounds) may actively modulate the tumour heterogeneity by exerting selective pressure on the evolving tumour and by dictating the genetic/epigenetic/phenotypic composition of the tumour [14]. So, it became crucial and urgent for biomolecular approaches to find novel biomarkers to improve early detection, diagnosis, monitoring and treatment prediction. The metabolites released from both cancer and stromal cells are essential part of the entire cancer secretome (mirroring the tumor microenvironment) and represent a reservoir of promising early and specific biomarkers detectable firstly in cancer-related biological fluids (like pleural, ascitic and breast fluids) and also circulating in blood as surrogate biomarkers [15].

### Unpromising and promising biomarkers to overcome tumour heterogeneity

The documented natural occurrence of heterogeneity in cancer cell populations within a tumor mass represents the major obstacle for finding both an early predictive biomarker and a successful therapeutic treatment [16].

A recent debate in the literature sheds light on the use-misuse-disuse in laboratory and clinical medicine of several cancer biomarkers, pointing out their difficulties to reach the clinic, and the reasons of different failure-success rates [17-20].

A biomarker (a little over 30 years old medical terminology) represents an indicator of a peculiar biological state that can objectively measure and compare normal biological and pathogenetic processes, or pharmacological responses to a therapeutic intervention [21]. Originating from tissues or body fluids, biomarkers may be potentially used as a risk factor and/or a useful tool to classify physio-pathologic conditions, to obtain basic informations underlying the pathogenetic mechanism(s) of human diseases, to detect cancers early, and to guide the choice of therapy [22]. The impact of biomarkers in laboratory and clinical medicine (especially in clinical oncology) is crucial to improve diagnosis, prognosis and treatment, in particular if the biomarker is detected before clinical symptoms or enables the monitoring of drug response [23,24].

Despite the frenetic bio-medical progresses (more than 570,000 publications on PubMed using “biomarker(s)” term, of which about 40% are “cancer biomarker”) and the substantial advances in the understanding of the molecular and bio-cellular basis of human diseases [25], a paucity of FDA-approved biomarkers is actually present [20]. Moreover, despite the great number of protein biomarkers described as promising candidates biomarkers, only few have been pursued to support clinical medicine, and most of them have unfortunately failed the validation studies [18,19].

The recent literature debate about cancer biomarkers has focused attention to several crucial aspects and caveats: a) complexity and underestimation of the problem, b) missing data on cancer biology knowledge, c) funding limitations, d) inappropriate clinical setting, e) unpromising discovery and scarce/neglected validation, f) pre-analytical and methodological shortcomings, etc. [18-20].

Starting from the evidence that cancer represents a cluster of multifaceted diseases involving alterations in both biomolecular pathways and multiple gene expression (regulating stemness, cell growth, survival, escape of immune surveillance, invasive and metastatic potential), the actual biological milestone of the cellular heterogeneity of tumors [4] represents the hardest obstacle for finding “ideal” protein cancer biomarker(s). Although a large number of candidate biomarkers have been individually discovered, only few promising combinations of them have been FDA-approved and are able to be translated into clinical practice [20].

A parallel effort is needed to characterize the heterogeneous composition of cancer cells and the influence of cancer microenvironment at both biochemical and molecular level. It shall also be crucial to detect and validate biomolecules as biomarkers, to provide diagnostic, prognostic or predictive informations [24].

It seems clear that not only intracellular proteins (the proteome), but also proteins secreted or shed into the tumor microenvironment (the secretome) may play crucial roles in driving the malignancy evolution of a tumor. In fact, the proteome reflects both the post-translational modifications and cellular pathways of a committed cancer cell; whereas improved secretome analyses provide insights into the mechanisms of cancer cells, the biology of tumor microenvironment, the cancer cell interactions and tumor progression.

The analysis of the cancer secretome represents a very promising approach able to detect cancer-related proteins directly in body fluids mirroring the tissue-specific tumor microenvironment, such as Nipple Aspirate Fluid (NAF) for breast cancer [15,26].

In fact, the secretome of NAF samples (noninvasively collected fluids from all breast cancer patients [27,28]) allow analysis of all the metabolites secreted by epithelial and stromal cells lining the breast ductal/lobular tree, representing the mirror breast microenvironment. The NAF secretome would represent a great opportunity for early diagnosis of breast cancer, limiting the decreased tumour-specificity of surrogate breast cancer biomarkers circulating in the blood.

### Cancer biomarkers in breast microenvironment secretome

Breast cancer is one of the leading causes of death among women around the world; due to its well known heterogeneity [29], encompassing multiple subgroups with different molecular signatures, prognoses, and responses to therapies, the exact molecular mechanisms underlying this multifaceted disease has yet to be fully elucidated. To obtain a significant reduction in morbidity and mortality in female breast cancer the main tool is the significant improvement of both conventional diagnostic techniques and laboratory methods to diagnose the disease earlier.

In fact, screening programs, digital mammography, specialized care and the widespread use of therapeutic agents, have reduced mortality rates but the identification of molecular targets remains a primary long-term goal for the development of specific early interventions and individual therapeutic strategies [30].

More than 10 million people are diagnosed with cancer every year, and it is estimated there will be over 15 million new cases/year by 2020 [31]. In particular, breast cancer (BC) is the most commonly diagnosed cancer among women, and screening through mammography and the early detection of disease has shown a significant mortality reduction in clinical trials [32].

In the past decade, there have been considerable improvements in the way that human breast tumours are identified and characterized, uncovering biomolecular alterations by using different technologies (like DNA assessment and mutation screening, gene-expression and microRNA, proteomic-metabolomic-degradomic profiling, etc.). In this respect, appropriate development of potential early detection and diagnostic tests, especially in the breast microenvironment, will be necessary prior to their clinical application, with special attention to their specificity and sensitivity to avoid over/under-diagnosis and/or clinical misuse [26,27].

The relevant biological role of breast microenvironment during cancer initiation and progression has been widely and continuously analyzed but unfortunately up-to-now not completely understood [33-35].

The microenvironment of the human mammary gland is composed of epithelial cells surrounded by intricate stroma (containing ECM components and various non-breast cells like fibroblasts, endothelial cells, and leukocytes). Through autocrine/paracrine mechanisms, physical and hormonal interactions result which are crucial for breast normal development and physiologic functions [36,37]. However, it is well known that alterations in these cell-cell and cell-matrix interactions (other than the single-cell biomolecular and epigenetic changes) may lead to the initiation and progression of BC [37,38].

Recently, the cell heterogeneity of a tumor mass and the interactions between tumor and microenvironment have been demonstrated (i.e., *solid tumors are not masses of equivalent cells, but instead contain cancer stem cells that support tumor maintenance*) [4,29]. Moreover, the composition of breast microenvironment may profoundly influence cellular phenotype, and drive tumor progression affecting disease outcome through diverse susceptibility to chemo-toxic insults [39,40].

The intraductal noninvasive approach of NAF secretome analyses may provide a panel of candidate biomarkers strengthening the armoury against BC [27,28]. All studies on the breast microenvironment have shown that the proliferation, survival, polarity, differentiation state, and invasive capacity of breast cancer cells can be modulated by myoepithelial and various stromal cells, mainly through signal molecules (such as growth factors, cytokines, glycosaminoglycans, proteases, hormones, etc.) involved in cellular pathways and paracrine regulatory networks [26].

Structural, cellular, functional and genetic alterations of stromal and epithelial cells may influence cell growth, morphogenesis and plasticity and contribute to the development of the tumorigenic phenotype [41].

Numerous studies have analyzed the expression of selected candidate biomarkers (both genes and proteins) in normal and neoplastic primary human breast tissues [42], up-regulation of invasion and angiogenesis related proteins and growth factors, may be involved in the gradual break down of the basal membrane separating epithelial and stromal cells [43,44].

These alterations can be monitored at the protein level and the protein signatures in the breast cancer microenvironment provide valuable information that may be an aid to more effective diagnosis, prognosis, and response to therapy, finally opening novel avenues for cancer-related biomarker discovery [30,45].

Knowledge about the breast and breast cancer microenvironments [26,36,37] is fundamental to identify and discern the pathologically different BC phenotypes [29,46], but also can help to perform personalized therapeutic strategies improving prognosis [24,47,48].

Many studies have found candidate biomarkers for early diagnosis and/or as possible reliable prognostic or predictive parameters, but in some cases contradictory results are reported [42,49]. The hot topic of cancer biomarkers has been recently debated [17,19-21,24]; among well-known biomarkers, uPA/PAI combination has long been regarded as prognostic indicator of BC, widely confirmed by prospective, retrospective and meta-analysis studies [49]. Nevertheless, uPA/PAI test has not been widely adopted in clinical practice, mainly linked to the fact that “*Clinicians usually prefer to over-treat some BC patients, instead of using prognostic biomarkers with less than perfect prediction*” [20].

#### The uPA-PAI system and the TF antigen

Among candidate biomarkers for human BC considerable attention has been focused recently on the combination of the *Urokinase-dependent plasminogen activator system* (uPA), *Plasinogen activator inhibitor* (PAI) and the *Thomsen-Friedenreich antigen* (TF).

The serine proteinase urokinase-type plasminogen activator (uPA; EC 3.4.21.73) and its specific inhibitors (the plasminogen activator inhibitor type-1 and −2, PAI-1 and PAI-2, respectively), are involved in the control of extracellular matrix turnover, tissue remodeling, cell adhesion and migration during physiopathological processes, including breast cancer [50,51].

Altered expression of the plasminogen activation system (uPA-PAI-uPA receptor) was found to be correlated with tumor malignancy. It is believed that the tissue degradation following plasminogen activation facilitates cancer cell invasion contributing to metastasis. Data accumulating over the past 20 years have shown that the uPA system shows multifunctional roles in healthy and neoplastic conditions [50]. In particular, the uPA system may affect breast cancer cell growth and its invasive and metastatic behavior (44); moreover, both uPA and PAI-1 have been associated with a poor prognosis in BC patients, predicting both outcome and response/resistance to specific therapies [52,53].

To date, these molecules are the only prognostic markers that have reached the highest level of evidence (LOE-1) in multi-centered clinical trials [52]. Unfortunately, the clinical utility of these molecules as biomarkers is limited to use as a laboratory test for basic cancer tissue detection and they are not yet used as prognostic tool [20,49].

The Thomsen–Friedenreich antigen (TF or CD 176), represents O-linked mucin type glycan found in about 90% of all human cancer and defined since 1920’s as pancarcinoma antigen [54,55].

Although the mechanism of increased TF expression occurrence in cancer is still not completely understood, its increasing expression on the cancer cell surface as the disease progresses may be mainly related to an active unmasking procedure linked to altered glycosylation mechanisms [56].

Although TF antigen roles in cancer are not completely understood (mainly due to different glycosylation patterns in different types of cancers), the pancarcinoma expression may have clinical utility as a potential immunotherapy target, leaving a possible glimmer of hope against recurrence of advanced BC [57].

### Early BC detection: new candidate biomarker combination on the horizon?

On the basis of the well known evidence that BC: 1) arises from the epithelial cells lining the ductal/lobular system, 2) is characterized by elevated cell heterogeneity, and 3) there is not currently a validated and FDA-approved BC combination of biomarkers; the secretome analyses of NAF fluids (as the mirror of the metabolic pathways and cellular modifications occurring in the breast microenvironment, both in physiological and pathological conditions [26]) may reveal early signs of precancerous and cancer transformation [27,58].

Starting from the first evidence of the clinical utility of NAF analyses dating back to 1950s [59], growing evidence has demonstrated and confirmed that this breast fluid is a rich source of candidate biomarkers for early diagnosis or risk assessment of BC [60,61]. All these studies have highlighted NAF as the optimal mirror of the breast microenvironment, in which ductal/lobular/stromal cell products, protein and hormonal components are secreted and/or accumulated during physio-pathologic conditions [27,28]. Also, among the wide number of bio-compounds present in NAF, proteinase analyses have gained increasing attention due to their role in the degradative balance into breast microenvironment at the tumor-host interface [62,63].

NAF samples thus represent the reservoir of biochemical and hormonal components secreted by the ductal tree appearing as the mirror of all metabolic changes occurring within the breast gland; whereas, plasma samples represent only the surrogate source which reflects only in part the tissue-specific metabolic changes. On these basis, the “intraductal approach” of the NAF secretome is more accurate, specific and timely for early breast cancer detection [64].

Due to the ever growing interest of molecular medicine moving from genomics to proteomics and metabolomics, the secretome of NAF represents a suitable method to discover BC specific biomarkers. So, NAF fluids represent protein-rich bioarchives highlighting what occurs within the microenvironment of the breast ductal system during all stages of the female breast life [61].

In the frame of searching cancer biomarkers in breast microenvironment [26,27], a recent study published in *BMC Cancer*[65] reports an interesting and innovative combinatory analysis of three well-known predictive biomarkers (uPA, PAI-1, and TF) in NAF collected noninvasively (or spontaneously secreted) from healthy women, patients with breast atypia and cancer (in pre- and post-menopause), requiring surgery because of a suspicious breast lesions.

Some previous studies have demonstrated that TF antigen was an independent predictor of disease only in post-menopasual women, correctly classifying cancer and atypia with a ROC value for disease prediction of 83% [66,67]. It has also been described that uPA system (uPA, uPAR, and PAI) represent useful independent predictors of cancer presence, providing both diagnostic and prognostic informations [68].

**Figure 1:**
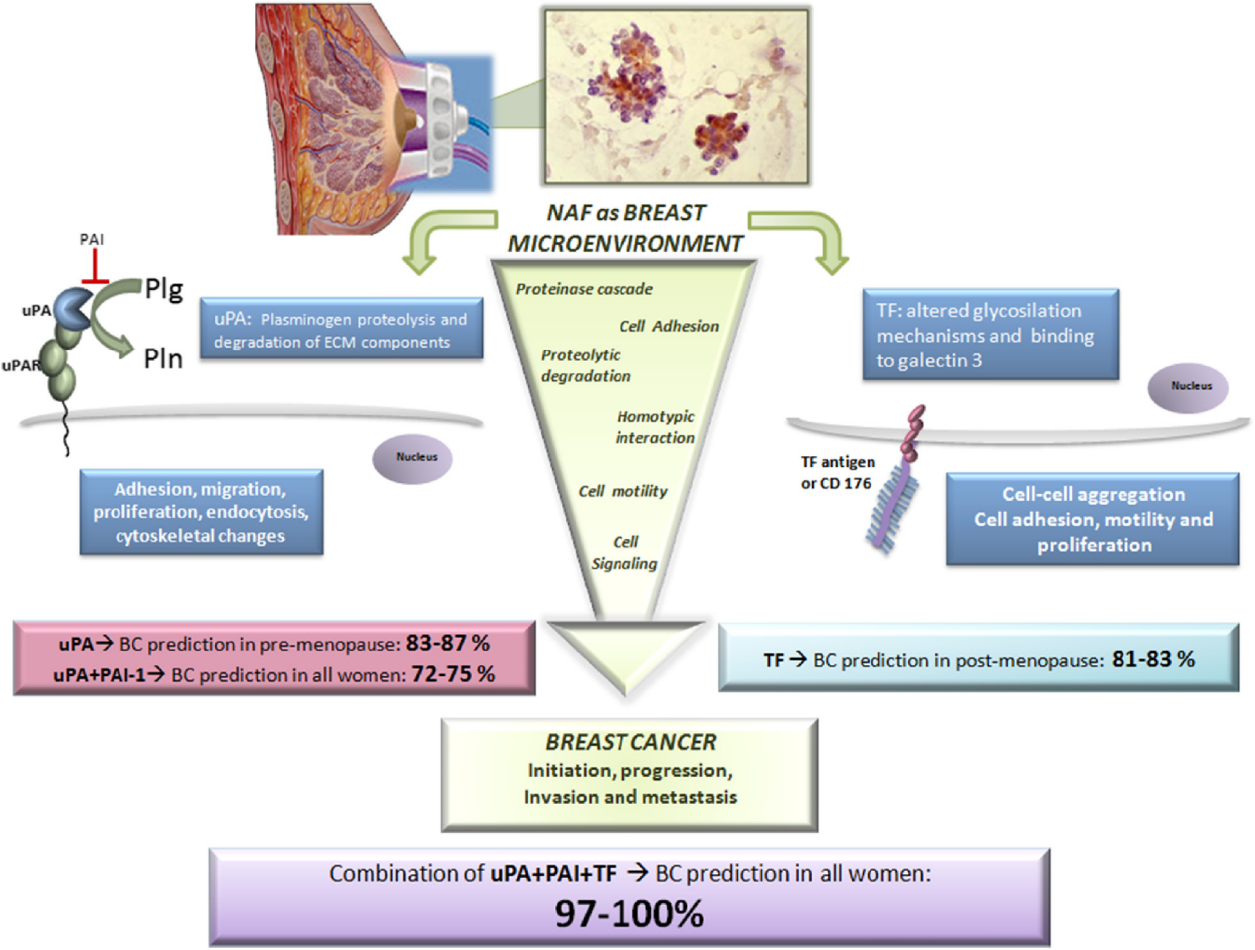
**Nipple Aspirate Fluid (NAF), secreted by non-lactating women into the breast ductal system, represents a mirror of the breast microenvironment.** NAF consists of secreted proteins and cells sloughed from stroma, ductal and lobular epithelium, containing several biomarkers that may be potentially useful tools. Several studies demonstrated implication of uPA-PAI system (on the left) and TF (Thomsen-Friedenreich) antigen (on the right) in several steps of breast cancer (BC) progression and metastasis through proteinase cascade activation, cell adhesion and motility. uPA (urokinase type Plasminogen Activator) is a serine protease whose main function is to catalyze the activation of plasminogen (Plg) into plasmin (Pln), after binding its receptor, uPAR (urokinase type Plasminogen Activator Receptor). Plasmin is able to degrade Extracellular Matrix (ECM), facilitating the release of several ECM components and proteolytic enzymes, leading to ECM remodeling and migration of BC cells. The uPA-PAI system activates signaling pathways promoting adhesion, proliferation and cytoskeletal changes in BC. The uPA physiological inhibition by PAI-1 (Plasminogen Activator Inhibitor 1) controls the activation of Plg into Pln and ECM degradation, modulating proliferation pathways. TF antigen (or CD 176) is a disaccharide constituted by the core 1 structure of O-linked mucin type glycans, which in normal cells is masked through glycosylation mechanisms. In tumors, various determinants can lead to alterations in O-glycosylation biosynthesis machinery, leading to the unmasking of TF antigen. Considering these candidate biomarkers individually, they provide incomplete prediction accuracy: in fact, uPA alone was predictive of breast disease only in premenopausal women (83–87%). TF alone was only predictive of breast disease in postmenopausal women (81–83%). It has been demonstrated that when TF+uPA+PAI-1 were combined, their predictive ability approached 100% allowing an excellent improvement of prediction of atypia or BC disease in women requiring surgery because of suspicious breast lesions.

Although these biomolecules appeared individually not perfectly accurate in predicting the presence of BC during different menopausal status, Sauter’s research team demonstrated the innovative biomarker combination in NAF [65]: 1) uPA concentration alone was more predictive of disease in premenopausal women (AUC values 0.83-0.87); 2) the range of AUC values for uPA+PAI-1 expression in all women was 0.72-0.75; 3) TF antigen alone was better at predicting BC in postmenopausal women showing range of AUC values 0.81-0.83. While TF antigen + uPA expression predicted breast diseases in both pre- and post-menopausal women with 84-92% of accuracy, interestingly and surprisingly when TF uPA + PAI-1 were combined, the predictive ability approached 97-100% allowing the near absolute prediction of both atypia or cancer disease in women requiring surgery because of a suspicious breast lesions (Figure 1).

Starting from the importance to simultaneously investigate multiple biomarkers in different breast diseases (like atypia and cancer) to avoid pitfalls, shortcomings and false discovery of candidate biomarkers [17,20], the multiple combination of well known biomarkers (TF, uPA and PAI-1) significantly contribute to the improvement of earlier diagnosis and prognosis of BC. So, Sauter’ study [65] is a promising example of how breast microenvironment biomarkers (that alone did not reach the excellence of the clinical/diagnostic/prognostic significance in pre and post-menopause, in pre-cancerous and cancer conditions) may be really useful only through a combination, providing predictive ability near to 1.0.

“*Viribus unitis*” (lat. "With united forces"): the intraductal noninvasive approach of NAF secretome analysis confirms the importance of considering biomarkers in the breast microenvironment not individually but in well validated combinations, to provide more informative diagnostic/prognostic tests and limiting the biomarkers included in the “*niche unmet needs*” [20].

## Conclusions

Breast cancer, the major cause of death among women around the world, is characterized by a high complexity and not completely understood biological and clinical heterogeneity [29]. Also by intricate interrelationships among the diverse cells composing the solid tumor mass (committed cancer cells, cancer stem cells, and non-cancer stem cells and non cancer cells) [4].

Moreover, the altered paracrine/autocrine mechanisms, physical, biomolecular and hormonal networks in the breast microenvironment lead to the development and progression of human BC [37], including the stemming tumor evolution and the capacity of breast cancer cells (through also cancer stem cell resistance) to support tumor maintenance also after an anticancer treatment [4].

The cell heterogeneity of BC reflects the complexity of the secretome (the mixture of hormones, proteins and proteinases that cancer mass is able to produce and secrete in the breast microenvironment), revealing the enormous difficulty to find useful and specific biomolecules as candidate cancer biomarker(s).

Improving technological methods (e.g., single-cell analysis) [10] to enable earlier detection and diagnosis of human BC in conjunction with the discovery and validation of a powerful combination of biomarkers, may represent the key tools to obtain a significant impact on morbidity and mortality in BC.

However, to effectively translate candidate biomarkers studies into the clinical setting, crucial factors have to be regarded (e.g., sensitivity and specificity of detection, ability to carry out quantitative measurements, standardization of sample collection, validation of the biomarker assay, clinical qualification of the biomarker, etc.) [21,24].

In this respect, the analysis of the breast cancer microenvironment, mirrored by NAF, allows the detection of alterations in biochemical, morphological and molecular pathways promoting cancer initiation, progression, invasion and metastasis, taking into account also the different stem/non-stem cell composition and interactions in the human breast microenvironment.

Therefore, analyzing NAF breast fluid proteins and proteinases (like TF, uPA and PAI) we could obtain useful insights about mechanisms making breast ductal cells more prone to morphological and biomolecular alterations (like migration/adhesion, proliferation and stem differentiation) [69].

Biomarker studies (including genetic, genomic, gene expression, proteomic and imaging approaches) may help the comprehension of disease initiation and progression, stem repopulation of cancer, prediction of patient population characteristics, finally improving also the critical points of drug development, drug efficacy and drug-induced adverse reactions.

It has been biologically and clinically demonstrated that individual biomarkers cannot predict or monitor cancer development/progression. To be highly effective in diagnostic/prognostic/clinical approaches it is crucial to combine specific biomolecules to obtain an optimal panel of biomarkers, which, through a comprehensive biomarker study registry, may significantly reduce false positives and hopefully identify promising cancer tests [70].

In this respect, the study recently published in *BMC Cancer* on TF, uPA and PAI in NAF samples [65] represents a shining example of how the combination of more biomolecules (alone not perfectly accurate) may significantly improve the prediction of breast atypia and/or cancer approaching near 100% accuracy, suggesting that they may be a useful breast cancer biomarker panel.

Future developments in onco-single-cell-omics [10] will potentially revolutionize cancer biology and clinical practice, allowing the identification of an ideal combination of biomolecules as reliable panel of biomarkers for the detection, diagnosis and monitoring of breast cancer [71].

## Abbreviations

BC: Breast cancer; uPA: urokinase-type plasminogen activator; uPAR: urokinase-type plasminogen activator receptor; PAI: Plasminogen activator inhibitor; ECM: Extracellular matrix; TF: Thomsen-Friedenreich carbohydrate antigen; CSC: Cancer stem cells; NAF: Nipple aspirate fluid.

## Competing interests

Both authors declare that they have no competing interests.

## Authors’ contributions

FM performed statistical analyses, drafted the manuscript and critically reviewed the final manuscript. DL performed database searches, drafted the manuscript and elaborated the figure. All authors read and approved the final manuscript.

## Pre-publication history

The pre-publication history for this paper can be accessed here:

http://www.biomedcentral.com/1471-2407/13/344/prepub
